# Narrative analysis in individuals with Parkinson’s disease following intensive voice treatment: secondary outcome variables from a randomized controlled trial

**DOI:** 10.3389/fnhum.2024.1394948

**Published:** 2024-05-22

**Authors:** Amy E. Ramage, Kathryn J. Greenslade, Kaila Cote, Jessica N. Lee, Cynthia M. Fox, Angela Halpern, Lorraine O. Ramig

**Affiliations:** ^1^Department of Communication Sciences and Disorders, Durham, NH, United States; ^2^Interdisciplinary Program in Neuroscience and Behavior, University of New Hampshire, Durham, NH, United States; ^3^LSVT Global, Inc., Tucson, AZ, United States; ^4^Teachers College, Columbia University, Communication Sciences and Disorders, New York, NY, United States

**Keywords:** Parkinson’s disease, vocal loudness, speech treatment, language, narrative discourse

## Abstract

Communication is often impaired in individuals with Parkinson’s disease (PD), typically secondary to sensorimotor deficits impacting voice and speech. Language may also be diminished in PD, particularly for production and comprehension of verbs. Evidence exists that verb processing is influenced by motor system modulation suggesting that verb deficits in PD are underpinned by similarities in the neural representations of actions that span motor and semantic systems. Conversely, subtle differences in cognition in PD may explain difficulty in processing of complex syntactic forms, which increases cognitive demand and is linked to verb use. Here we investigated whether optimizing motor system support for vocal function (improving loudness) affects change in lexical semantic, syntactic, or informativeness aspects of spoken discourse. Picture description narratives were compared for 20 Control participants and 39 with PD, 19 of whom underwent Lee Silverman Voice Treatment (LSVT LOUD®). Treated PD narratives were also contrasted with those of untreated PD and Control participants at Baseline and after treatment. Controls differed significantly from the 39 PD participants for verbs per utterance, but this difference was largely driven by untreated PD participants who produced few utterances but with verbs, inflating their verbs per utterance. Given intervention, there was a significant increase in vocal loudness but no significant changes in language performance. These data do not support the hypothesis that targeting this speech motor system results in improved language production. Instead, the data provide evidence of considerable variability in measures of language production across groups, particularly in verbs per utterance.

## 1 Introduction

Language and movement are intertwined. This is true when children learn words and rules about their language, and it remains true for the moment-to-moment formulation of sentences in verbal communication (Wang et al., 2019). Voice and speech are the components of the motor system that are the vehicles on which oral language is expressed. It stands to reason, then, that the neural systems underlying movement and those underlying language production may be intertwined. However, motor and language systems are frequently considered separately, particularly when approaching remediation of either.

An example of this is in individuals with Parkinson’s disease (PD) who have characteristic impairments in vocal motor control secondary to dopaminergic deficiencies (Hallett and Khoshbin, 1980; Berardelli et al., 2001; Fox et al., 2002). PD results in hypokinetic dysarthria in up to 89% of patients (Logemann et al., 1978; Hartelius and Svensson, 1994; Ho et al., 1998; Miller et al., 2007; Schalling et al., 2017), affecting vocal quality [i.e., reduced loudness, hoarse and/or breathy quality, monotone prosody (Fox and Ramig, 1997; Ho et al., 1998; Baumgartner et al., 2001; Midi et al., 2008; Plowman-Prine et al., 2009)] as well as speech clarity (i.e., articulatory precision, vowel centralization) and speech rate (Logemann and Fisher, 1981; Forrest et al., 1989; Ackermann and Ziegler, 1991; Tjaden and Wilding, 2004; Sapir et al., 2007; Skodda and Schlegel, 2008; Sapir et al., 2010; Tjaden et al., 2013).

Language is also altered in individuals with PD. Action word or verb processing is diminished (Péran et al., 2009; Rodríguez-Ferreiro et al., 2014; Salmazo-Silva et al., 2017; Johari et al., 2019), particularly for those that are associated with an individual’s location of greatest motor impairment[i.e., upper vs. lower limbs; (Roberts et al., 2017)]. Recognition, comprehension, or production of highly actionable nouns (e.g., screwdriver, toothbrush) is represented in the brain across semantic and motor domains (Noppeney et al., 2006), but is minimally impaired in PD, if at all (Bocanegra et al., 2017). Differences in action word processing tend to be verb-specific, are seen early in the PD pathogenesis, and affect access to lexical representations for the comprehension and production of action words in isolation and in contextual interactions [e.g., (Cardona et al., 2013)]. However, verb processing in individuals with PD is not necessarily associated with motor impairment severity (D’Ascanio et al., 2023), unlike in other motor disorders [e.g., amyotrophic lateral sclerosis (ALS) in (Cousins et al., 2018); cervical dystonia in (Bayram and Akbostanci, 2018)]. Nonetheless, there may be a unique interaction between the motor and language systems in PD, likely centered in cortico-striatal loops (Silveri et al., 2012; Cardona et al., 2013). Investigations of several motor disorders have parsimoniously localized aspects of action concepts to cortico-striatal loops [as impaired in individuals with PD (Cardona et al., 2014)], rather than to primary motor or premotor cortex [as is impaired in individuals with ALS, (Cousins et al., 2018)]. The latter finding highlights a potential for effector-specific representations of the semantics for action concepts, a hypothesis that is best couched in the theories of embodied cognition (Pulvermüller, 2005; Barsalou, 2010; Binder and Desai, 2011; Barsalou, 2020).

What remains unclear is whether action word or verb processing in PD is attributable to a motor system impairment influencing language processing specifically, or if it is related to the presence of cognitive impairment (Murray, 2000; Bocanegra et al., 2017; Salehi et al., 2024). Subtle cognitive differences are observed in PD, particularly in executive functioning and processing speed (Schapira et al., 2017; Smith and Caplan, 2018), which could explain impaired language in contexts of higher processing load like discourse. Thus, observations of action word differences for PD may be attributable more generally to the cognitive deficits influencing sentence processing and the executive planning and working memory demands for syntactically complex stimuli.

One approach to investigating the motor system and its relationship to language processing in PD is through observation of performance changes when individuals are on or off dopaminergic medications. Individuals with PD on dopaminergic medications produce more action verbs and action content in verbal fluency tasks compared to those not taking their medications (Herrera et al., 2012). Herrera and colleagues concluded that “the dopamine network from basal ganglia to brain motor areas might play a role in retrieving action verbs with specific semantic representations” (p. 72). This would suggest that the deficit influences the early stages of message formulation, at the level of lexical semantics (Ferreira, 2000), but is also likely influenced by the task employed (e.g., verbal fluency for which lexical retrieval is the target behavior).

A similar approach to investigating dopaminergic influence on language has been used to investigate individuals with PD who have undergone surgical implantation of deep brain stimulators. A small number of studies of language performance assessed pre- and post-surgical stimulator placement in the subthalamic nucleus (Zanini et al., 2003) or the pedunculopontine nucleus (Zanini et al., 2009) have reported improvements in morphosyntax with stimulator on versus off. That is, word structure (morphology) and grammar (syntax), both of which are thought to be guided by verb selection (Ferreira, 2000), improved with stimulation. Despite the small sample sizes, no changes (good or bad) were observed pre- to post-surgery or on/off stimulation for neuropsychological measures or lexical semantics (words and meanings), suggesting discourse production effects were specific to later stages of utterance planning – i.e., surface structure for grammatical encoding, function assignment, or constituent assembly (Bock and Levelt, 1994; Ferreira, 2010).

These findings suggest that language production may vary with modulation of the motor system in individuals with dopaminergic degeneration. A potential extension of this theory is that improving motor system function could improve language function in PD. The current study aims to determine whether a treatment targeting improved motor (voice) function in PD is associated with concomitant improvements in verbal language production, specifically in morphosyntax. This study is a secondary analysis of narrative language data from a randomized controlled trial of the Lee Silverman Voice Treatment (LSVT LOUD®). This intervention targets change at the level of motor function (respiratory-laryngeal-oromotor support) and has been shown to effectively improve vocal loudness in individuals with PD (Ramig et al., 2018; Perry et al., 2024) as well as other areas of speech motor function and communication not targeted, including speech articulation (Sapir et al., 2007), intonation (Ramig et al., 2001), speech intelligibility (Levy et al., 2020; Schulz et al., 2021), facial expression (Dumer et al., 2014), swallowing (El Sharkawi et al., 2002; Miles et al., 2017), and neural functioning related to speech (Narayana et al., 2010; Baumann et al., 2018). Given these documented system-wide improvements, this intervention might also effect changes in language production. Thus, the aims of this study are to (a) replicate previously reported differences in discourse-level language production and information content in individuals with PD relative to neurotypical, age-matched healthy controls, and (b) determine whether participation in LSVT LOUD associates with improvements in discourse-level language production or information content. We hypothesize that differences in discourse-level language production may be observed between individuals with PD and controls at Baseline as found in previous studies. Further, we hypothesize that the intervention may yield improvements in discourse-level language. If such improvements are specific to verb production – either in terms of retrieval or use of verbs in context (morphosyntax), this result may lend support to the idea that motor and language domains are intertwined.

## 2 Methods

Participants are those reported in the LSVT LOUD protocol (Ramig et al., 2018). Participants with PD were diagnosed by a neurologist, were between stages I to IV on the Hoehn and Yahr scale for symptom severity (Hoehn and Yahr, 1967) and were stable on antiparkinsonian medications. The PD participants’ levodopa equivalence is reported in (Ramig et al., 2018). All were 45–85 years of age with normal hearing for age, mental status [scored ≥25 on the *Mini-Mental Status Examination* (MMSE, Folstein et al., 1975)], and reported no greater than moderate symptoms of depression [based on the *Beck Depression Inventory II* ≤ 24, (Beck et al., 1996)]. Individuals were excluded if they had PD with other neurological conditions or atypical PD at the time of screening, presented with speech or voice disorders unrelated to PD, had undergone neurosurgical treatment, had laryngeal pathology or surgical history, or had swallowing impairment requiring treatment. Additionally, individuals with PD who had undergone intensive speech treatment within the 2 years prior to the study or who had participated in LSVT LOUD previously were excluded.

Study procedures were approved by the institutional review board at the University of Colorado at Boulder and University of Colorado Health Science Center and are shared at ClinicalTrials.gov identifier: NCT00123084. All participants provided informed written consent for participation. Please see Ramig et al. (2018) for details regarding screening and randomization for the LSVT LOUD treatment trial.

Twenty participants with idiopathic PD underwent LSVT LOUD (TXPD) for the full 4-week intensive dose. Participants received 16, one-hour sessions of voice treatment, delivered 4 days a week over 4 weeks (see details in Supplementary materials). From the 22 TXPD and 22 UNTXPD participants reported in Ramig et al. (2018), three were later found to have MSA or palilalia and were excluded from this study. Additionally, one TXPD participant was an outlier for several language variables (i.e., words per minute, MLU words, verbs per utterance, density, and content units), and thus his data were removed from the study, leaving a total of 19 TXPD participants. A second group of 20 participants with idiopathic PD served as an untreated comparison PD group (UNTXPD), and 20 age-matched, neurotypical participants served as healthy controls (Controls).

### 2.1 Picture description and dB SPL data acquisition

A range of speech tasks were administered at Baseline (Baseline, pre-intervention in the TXPD group) and after 4-weeks (one-month, immediately post-intervention in the TXPD group). This study focused on one picture description task, the Cookie Theft picture [*Boston Diagnostic Aphasia Examination*; (Goodglass et al., 2001)]. Participants were asked to describe the Cookie Theft picture in as much detail as they could for about a minute. Audio files were edited to eliminate coughs, extraneous talking not related to the task, etc. (e.g., requests to the clinician). The edited, calibrated microphone files were analyzed for SPL resulting in a mean and standard deviation value for dB SPL at a reference distance of 30 cm, providing a metric of loudness.

### 2.2 Transcription, segmentation, and coding procedures

Language samples were transcribed using CHAT conventions in the CLAN program [V 30-Jan-2020, (MacWhinney, 2000)]. Transcripts were separated into conversational (c-) units, defined as a main clause and all dependent clauses (Loban, 1976). The end of an utterance was marked by use of: (1) a coordinating conjunction (*for*, *and*, *nor*, *but*, *or*, *yet*, *so*) connecting two main clauses; (2) terminal intonation contour; or (3) a complete c-unit (main clause and all attached dependent clauses).

Two transcribers (KC and JNL), naïve to participant group, time point, and other identifying information, were trained with six samples from the current data set to establish reliability. Next, they independently transcribed an additional 10 samples to confirm that interrater reliability reached sufficient levels (>0.80 for intraclass correlation coefficient (ICC) and point-to-point agreement). At this point, the remaining 104 samples from the data set were transcribed in four blocks to monitor reliability and prevent drift. Then, EVAL (MacWhinney, 2000), FLUCALC, and frequency of verbs were run to obtain scores for various language variables, fluency, and verb analysis, respectively. The accuracy of CLAN’s verb coding was checked (KC). Due to a verb coding error rate of 7.46% (e.g., “socks” coded as a verb), three graduate students checked all verbs for coding accuracy, achieved consensus, and re-coded as needed to reflect the correct part of speech.

Thirty-four language samples (29.82%) were checked for reliability. For each sample, a two-way random ICC with absolute agreement was computed [SPSS version 26.0.0.0, (IBM Corporation, 2015)]. In addition, point-to-point reliability for utterances (91.59%) and words (94.55%) was calculated for thirty samples. Disagreements were discussed and resolved by consensus. As summarized in Supplementary Table S1, ICCs for the variables derived from EVAL and FLUCALC ranged from moderate (0.50–0.75) to excellent (> 0.90).

### 2.3 Discourse information analysis coding procedures

Main concepts and content units were coded manually (KC and JNL). Main concepts were coded for the presence, accuracy, and completeness of seven statements that define the essential elements for the Cookie Theft picture [(Nicholas and Brookshire, 1995), coding procedures and reliability metrics in Supplementary materials]. Once interrater reliability was established, coding proceeded in four blocks to monitor reliability and prevent drift. For main concepts, 24 of 99 samples (24.2%) were checked for reliability, yielding an average *k* = 0.809 and point-to-point reliability of 82.2%. For content units, 27 of 114 samples (23.7%) were checked for reliability, yielding an average *k* = 0.929 and point-to-point reliability of 92.9%. Consensus data were created following reliability analyses and used for study analyses.

The following language variables served as indicators of:

- Fluency and efficiency: words per minute, total number of utterances produced, and mean length of utterance (MLU).
- Syntax: inclusion of verbs to form complete utterances and the syntactic complexity of those utterances were indexed by the number of verbs per utterance (VPU).
- Lexical-Semantic: vocabulary diversity/variation was indexed by type-token ratio (TTR), calculated as the number of unique words spoken (types) divided by the total number of words produced (tokens). Propositional idea density, an index of a speaker’s assertions as opposed to references to entities (Brown et al., 2008), was calculated as the total number of verbs, adjectives, adverbs, prepositions, and conjunctions divided by the total number of tokens.
- Informativeness: Main concepts and content units as coded and described above.

### 2.4 Data analysis

Because the current study represents secondary analyses of an RCT, the study was powered for the original study’s planned analyses. The present study’s language outcomes included 6 dependent variables (number of utterances, MLU, VPU, TTR, propositional idea density, main concepts, and content units), while the original study had one (vocal loudness in dB SPL). The G*Power 3.1.9.4 software package was used to re-calculate power (Faul et al., 2007), including the 10 dependent variables and 3 groups. The total sample size of n = 60 provided 84% power to detect a large effect size (*f^2^* = 0.35) and 60% power to detect a medium effect size (*f^2^* = 0.15) for assessing group differences at Baseline and differences between assessment timepoints by group. All variables were assessed for outliers and distribution normality (Shapiro–Wilk test) in SPSS. Several of the language variables were non-normally distributed, and there was considerable within-group variance (Supplementary Table S2).

Depending on normality results, general linear models (GLMs) or Mann Whitney U-tests were used to analyze group differences in demographic variables. Pearson or Spearman bivariate correlations identified the interrelationships between (1) loudness (in dB SPL) and (2) the discourse variables with significance criterion at *r* or *rho* > |0.30|, *p* < 0.01.

#### 2.4.1 Quantile linear mixed effects modeling

Quantile linear mixed effects modeling (LQMM) offered a nuanced way to analyze data without the strict assumption of normality (Liu and Bottai, 2009; Geraci and Bottai, 2014). This modeling is a fusion of linear mixed effects models and quantile regression. Linear mixed effects models (LMM) are a versatile tool for modeling data with hierarchical or correlated structures, like those in the present study. They incorporate both fixed effects (like traditional linear regression) and random effects, which account for the data’s hierarchical nature. Random effects capture variations between different groups or clusters in the data, making LMMs suitable for studying data with dependencies such as data derived from longitudinal or pre/post designs like this one. Quantile regression extends the conventional linear regression by modeling various quantiles (e.g., median, lower, and upper percentiles) of the response variable providing insights into how predictor variables influence different parts of the response distribution, making it robust against outliers and skewed data. LQMM leverages LMM’s ability to handle hierarchical data structures and quantile regression’s flexibility to model different parts of the response distribution. This allows for analysis of the relationship between predictors and the response variable across multiple quantiles while considering the data’s inherent complexity. As well, LQMM is resilient to non-normality in the data. Interpreting model results involves examining how predictor variables affect different quantiles of the response variable. This approach provides a more comprehensive understanding of the relationships, especially when these effects vary across quantiles. Further, LQMM is robust to outliers, making outlier detection and removal unnecessary when employing quantile linear mixed effects models. Generally, for interpretation purposes, the 50^th^ quantile (i.e., median regression or robust regression) is the primary focus of such an analysis, but analyses are reported for the 10th, 25th, 50th, 75th, and 90th quantiles. All tests were conducted with a false discovery rate (FDR) correction for multiple comparisons. All tests were conducted in R using the lqmm package.

## 3 Results

### 3.1 Participant demographics and descriptive statistics at Baseline

The PD and Control groups did not differ for age (*U* = 421, *p* = 0.62), gender (*X^2^_1_* = 0.58, *p* = 0.45) or handedness (*X^2^_4_* = 2.98, *p* = 0.56) distribution, or for *Mini-Mental Status Exam* score (*U* = 303.5, *p* = 0.14) (Table 1) (Folstein et al., 1975). The PD group endorsed more symptoms of depression on the *Beck Depression Inventory II* (BDI, Beck et al., 1996) (*U* = 641, *p* < 0.001), with three participants scoring above the clinical cutoff of 17. The treated and untreated PD groups did not differ for BDI score (*U* = 148, *p* = 0.25), motor symptom severity (Hoehn & Yahr scale, (Bhidayasiri and Tarsy, 2012), *U* = 165, *p* = 0.50) or years since diagnosis (*U* = 209.5, *p* = 0.59). It was reported in (Ramig et al., 2018) that the TXPD and UNTXPD participants did not differ significantly for levodopa equivalence. Supplementary Table S2 presents the descriptive statistics by group for the variables of interest in the study.

**Table 1:** Subject demographics and symptoms.

	TXPD	UNTXPD	Control
n	19	20	20
Age	67 ± 8	65 ± 9	64 ± 9
Gender (M:F)	15:4	13:7	13:7
Handedness (R:L)	17:1	16:4	19:1
Beck Depression Inventory II*	9.47 ± 6	7.3 ± 5	2.85 ± 3
Mini-Mental Status Exam	28.8 ± 1	29.0 ± 1	29.3 ± 1
Hoehn & Yahr Scale	2 ± 0.8	2.03 ± 1	-
Years Since Diagnosis	4.83 ± 7	4.73 ± 4	-

^*^TXPD = UNTXPD > Controls, *p* < 0.001.

Correlations amongst the study variables (Supplementary Table S3) indicated that number of utterances correlated positively with content units [*rho* (59) = 0.45, *p* < 0.001] and inversely with verbs per utterance [*rho* (59) = −0.50, *p* < 0.001], MLU [*rho* (59) = −0.49, *p* < 0.001], and TTR [*rho* (59) = −0.57, *p* < 0.001]. TTR inversely correlated with content units [*rho* (59) = −0.52, *p* < 0.001]. VPU correlated with MLU [*rho* (59) = 0.62, *p* < 0.001] and propositional idea density [*rho* (59) = 0.38, *p* = 0.003], and MLU correlated with propositional idea density [*rho* (59) = 0.40, *p* = 0.002]. Finally, words per minute correlated positively with number of utterances [*rho* (59) = 0.63, *p* < 0.001] and content units [*rho* (59) = 0.60, *p* < 0.001], and inversely with TTR [*rho* (59) = −0.59, *p* < 0.001].

Given the non-normal distributions and collinearity, the variables were evaluated with LQMM to assess how, on average, the TXPD, UNTXPD and Controls changed over time, from Baseline to one-month assessments. Descriptive data for these two time points are provided in Supplementary Table S4. Then, a second model was run to determine if either loudness or rate (words per minute) significantly influenced the assessed Baseline values or changes across groups. In both cases, the 10th, 25th, 50th, 75th, and 90th quantiles were considered. Table 2 summarizes estimates for each variable across quantiles.

**Table 2:** Parameter estimates for each variable across quantiles for each group at Baseline and 1-month.

			Quantile				
Outcome	Group	Visit	0.10	0.25	0.50	0.75	0.90
Loudness (dB SPL)	Control	Baseline	68.70	69.60	71.70	72.90	74.30
	Control	Follow-up	68.50	69.80	70.80	72.80	74.70
	TXPD	Baseline	64.20	68.10	69.90	72.40	73.30
	TXPD	Follow-up	71.10	73.50	74.90	77.20	78.20
	UNTXPD	Baseline	64.80	67.90	69.40	70.40	73.00
	UNTXPD	Follow-up	65.00	66.70	70.40	72.10	74.00
Words per minute	Control	Baseline	112.13	119.09	135.48	148.29	158.03
	Control	Follow-up	110.48	121.82	133.33	146.77	159.71
	TXPD	Baseline	91.07	108.39	129.64	138	170.48
	TXPD	Follow-up	108.18	125.58	138.81	150.45	162.73
	UNTXPD	Baseline	98.44	110.53	132.73	151.77	175.91
	UNTXPD	Follow-up	103.00	110.00	136.55	155.81	189.15
Number of utterances	Control	Baseline	10.82	12.80	14.30	15.80	17.50
	Control	Follow-up	9.71	11.50	13.20	14.50	16.30
	TXPD	Baseline	9.97	10.40	11.90	14.00	16.40
	TXPD	Follow-up	10.41	10.30	11.60	13.50	16.80
	UNTXPD	Baseline	8.31	10.30	12.30	14.50	15.30
	UNTXPD	Follow-up	9.91	11.40	13.60	15.50	15.40
Mean length of utterance	Control	Baseline	7.90	9.01	9.85	11.30	12.00
	Control	Follow-up	8.40	9.57	10.77	12.40	12.80
	TXPD	Baseline	6.82	8.31	9.56	11.60	11.90
	TXPD	Follow-up	7.48	9.27	10.85	13.00	13.40
	UNTXPD	Baseline	8.77	9.60	10.61	11.90	12.80
	UNTXPD	Follow-up	8.58	9.11	10.42	11.10	11.30
Type-token ratio	Control	Baseline	0.56	0.55	0.53	0.55	0.55
	Control	Follow-up	0.56	0.55	0.52	0.55	0.53
	TXPD	Baseline	0.53	0.49	0.53	0.55	0.58
	TXPD	Follow-up	0.53	0.48	0.53	0.55	0.59
	UNTXPD	Baseline	0.56	0.52	0.54	0.59	0.59
	UNTXPD	Follow-up	0.55	0.53	0.54	0.58	0.56
Verbs per utterance	Control	Baseline	1.18	1.25	1.42	1.64	1.82
	Control	Follow-up	1.18	1.25	1.42	1.66	1.87
	TXPD	Baseline	1.12	1.18	1.44	1.65	1.96
	TXPD	Follow-up	1.18	1.38	1.59	1.88	2.32
	UNTXPD	Baseline	1.45	1.57	1.80	2.03	2.17
	UNTXPD	Follow-up	1.33	1.46	1.55	1.69	1.80
Propositional idea density	Control	Baseline	2.41	2.40	2.40	2.36	2.41
	Control	Follow-up	2.34	2.32	2.42	2.35	2.35
	TXPD	Baseline	2.42	2.39	2.40	2.40	2.45
	TXPD	Follow-up	2.30	2.34	2.31	2.33	2.30
	UNTXPD	Baseline	2.25	2.25	2.25	2.25	2.25
	UNTXPD	Follow-up	2.31	2.31	2.31	2.31	2.29

#### 3.1.1 Fluency and efficiency

The number of utterances produced and words per minute did not differ significantly between the three groups at Baseline or at the one-month follow-up. Controls producing more words per minute had significantly higher numbers of utterances across all quantiles at Baseline (Supplementary Table S4).

#### 3.1.2 Lexical semantics

The groups did not differ for TTR values at Baseline or 1-month, but TXPD individuals with a higher number of words per minute had significantly higher Baseline TTR values for the 90^th^ quantile. No significant differences across the different groups were found for propositional idea density, nor did loudness or words per minute influence TTR or propositional idea density across all quantiles studied.

#### 3.1.3 Syntax

**Figure 1:**
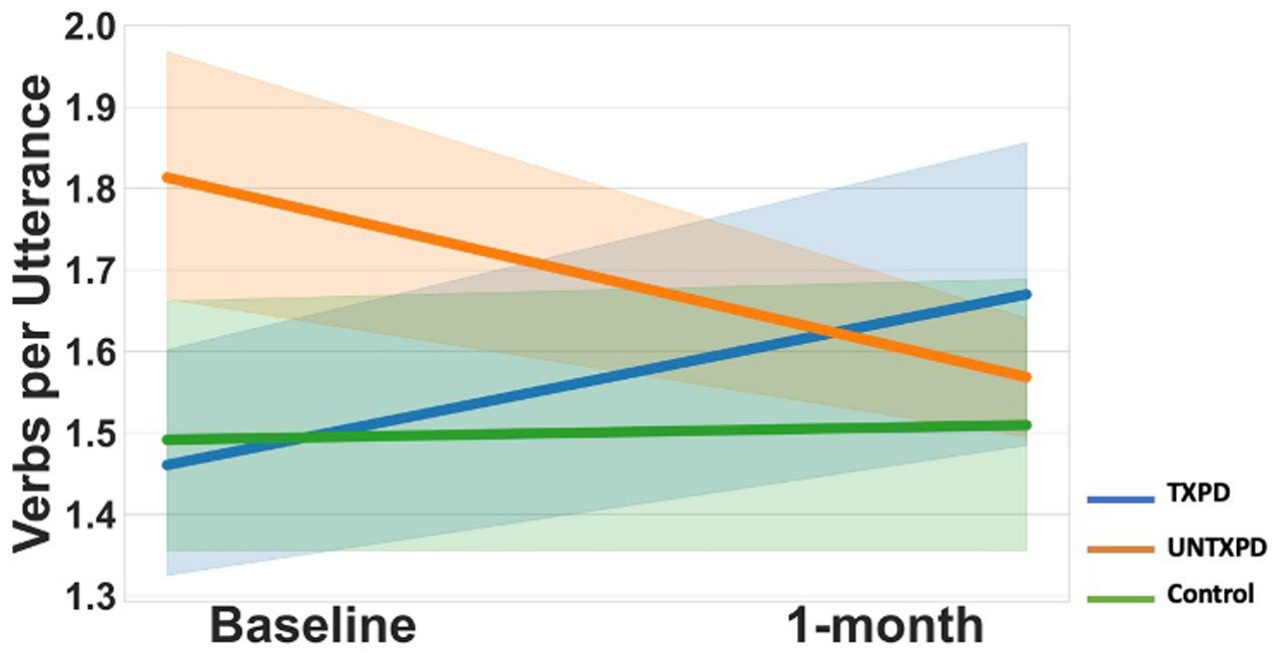
Multiple differences across groups existed for verbs per utterance, generally reflecting little change over time in the Controls, decrease over time in the UNTXPD, and increase over time in the TXPD group. However, given the considerable variance in this measure, the only significant change was the decrease in VPU observed in the UNTXPD group.

**Figure 2:**
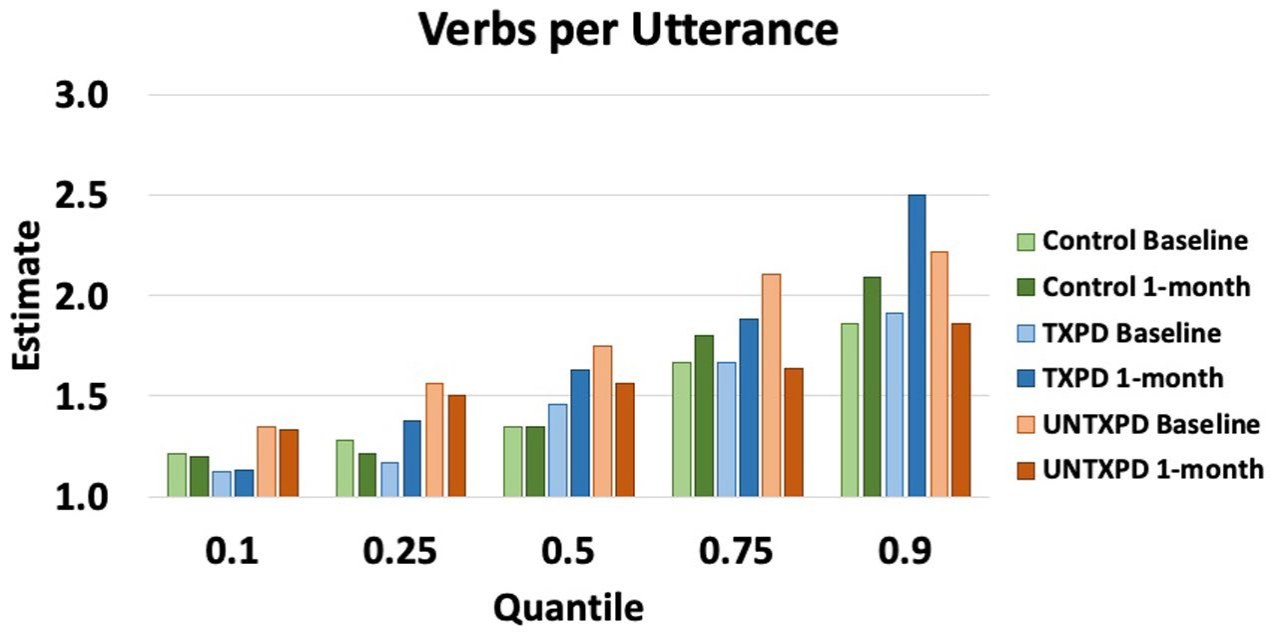
Estimates for verbs per utterance demonstrate considerable variability in verbs per utterance in the PD participants across quantiles, with the UNTXPD producing significantly more verbs per utterance than either of the other groups at Baseline, but those significantly decreased at 1-month for the 50th, 75th, and 90th quantiles.

VPU demonstrated multiple significant differences across groups (Figures 1, 2). First, the UNTXPD group had significantly higher Baseline values than the Control (10th, 25th, 75th, and 90th quantiles) and TXPD (10th and 25th quantiles) groups. The UNTXPD group significantly decreased in VPU over time (Baseline >1-month for the 75th and 90th quantiles) while there was no significant change in the TXPD or Control groups. Note however that there was a considerable but nonsignificant increase in VPU in the TXPD group post-intervention (Figure 2) for those in the 90th quantile. Controls in the 25th quantile with higher words per minute produced more VPU. Neither loudness nor words per minute influenced VPU in the PD groups.

## 4 Discussion

The present study tested whether linguistic information produced during the BDAE Cookie Theft picture description task differed in individuals with PD compared to age-matched Controls. The study also assessed whether completion of an intensive intervention targeting increased vocal loudness might improve the motor system engagement for speech and, in turn, strengthen access to action concepts, improving retrieval or production of verbs and syntactic complexity in these narratives. Elicited from a convenience sample of treated and untreated participants with PD along with age-matched controls from a randomized controlled trial of LSVT LOUD, these narratives were used to test whether (1) lexical semantic or morphosyntactic language variables differed in the PD groups compared to Controls at Baseline, and (2) whether treatment-related change in vocal loudness (i.e., motor system) corresponded with changes in lexical semantic or morphosyntactic language production.

### 4.1 Group differences in narratives

At Baseline, participants with PD did not differ from Controls for lexical semantics on the picture description task, as measured by lexical diversity (TTR), propositional idea density, or informativeness as measured by content units and main concepts. The only significant difference was for VPU, which varied considerably among PD participants. Specifically, the UNTXPD group produced more VPU than the Control or TXPD groups at Baseline, particularly when considering the lowest and highest quantiles (Figure 2). Qualitative characterization of productions across groups indicates that the difference in VPU may relate to the nature of the task rather than to a true difference in verb production. That is, picture descriptions yield variable performance with some individuals listing items seen in the picture (i.e., producing no verbs); others producing few utterances but using complete, grammatically correct sentences; and most doing both (e.g., “boy, and girl, the boy is falling”). In this cohort, several PD participants produced few utterances, but each included a verb, inflating the median VPU. Other tasks that have identified verb-specific deficits in PD include production of verbs in isolation [e.g., verb naming, (Bocanegra et al., 2015)] and lexical decision-making or semantic association tasks, which rely on verb recognition or comprehension [e.g., (Bocanegra et al., 2015; Roberts et al., 2017)] rather than production of verbs in context. These task-related factors could help explain why the current study’s narrative task did not replicate previous findings of differences in verb use. Regardless, the current findings suggest that verb production differences in participants with PD are not attributable to lexical semantics or morphosyntax.

Narrative samples from participants with PD tended to include fewer utterances with somewhat reduced propositional idea density and reduced lexical diversity (TTR), though these differences were not significantly different from that of Controls. Nonetheless, these findings are consistent with previous studies (Murray, 2000; Murray and Lenz, 2001) – participants with PD generally say less and produce syntactically simple utterances. These differences in morphosyntactic aspects of language production are in the latter stages of processing for production (Bock and Levelt, 1994; Ferreira, 2010) and suggest little PD-related difference in word access and retrieval.

### 4.2 Change in the TXPD group given intensive voice treatment

VPU was the only language variable to demonstrate change over time in this study. On average, VPU significantly decreased in the UNTXPD group, while the TXPD group demonstrated considerable (though nonsignificant) increases in VPU between time points. The only covariate associated with this change was words per minute, with participants in all groups producing more VPU if they produced more words per minute. Notably, there was no significant change in words per minute for any group. As noted above, VPU was variable across participants in all groups at both time points, with variability likely reflecting task characteristics.

To our knowledge, there is only one previous study that investigated language production change in participants with PD following a motor-based behavioral intervention. Altmann and colleagues (Altmann et al., 2016) compared sentence-level picture description performance (producing one sentence to describe a single action depicted in a picture without using pronouns) in participants with PD before and after either an aerobic exercise program or a stretch-balance program. The sentences were analyzed for completeness (including characters and verbs), fluency (pauses or false starts), and grammatical accuracy. These authors hypothesized that aerobic training, which had been shown previously to improve cognition and memory, would improve cognition and executive function, and in turn improve language. They found that aerobic training in the PD group improved completeness, but there were no significant changes in fluency or grammaticality. That study did not assess change in motor system function to determine whether one of the training types was more or less likely to improve motor function. Nonetheless, these findings were consistent with current results, namely that verb use in utterances/sentences increased with motor training, but not to a significant extent.

In summary, current results did not fully support our central hypothesis that improving motor system function through vocal practice might influence the representations of action words, in turn improving access to verbs. Individuals with PD who underwent an intensive voice treatment to increase vocal loudness had concomitant increases in verbs per utterance, but that increase was not greater than this metric’s variance across groups. The verb is the core of sentence production with morphology and syntax falling in line after the verb is selected. Had the increase in verbs per utterance in the treated PD participants also led to use of more morpho-syntactically complex sentences - i.e., more lexically dense utterances, then the relationship between motor and language systems might have been established. However, that was not the case in this study.

## Data availability statement

The original contributions presented in the study included in the article/Supplementary material, further inquiries can be directed to the corresponding author.

## Ethics statement

Study procedures were approved by the institutional review board at the University of Colorado at Boulder and are shared at ClinicalTrials.gov identifier: NCT00123084. All participants provided informed written consent for participation. Please see Ramig et al. (2018) for details regarding screening and randomization for the LSVT LOUD treatment trial. The studies were conducted in accordance with the local legislation and institutional requirements. The participants provided their written informed consent to participate in this study. Written informed consent was obtained from the individual(s) for the publication of any potentially identifiable images or data included in this article.

## Author contributions

AR: Conceptualization, Data curation, Formal analysis, Methodology, Project administration, Supervision, Validation, Visualization, Writing – original draft, Writing – review & editing. KG: Formal Analysis, Methodology, Resources, Writing – original draft, Writing – review & editing. KC: Methodology, Writing – original draft, Writing – review & editing. JL: Methodology, Writing – original draft, Writing – review & editing. CMF: Conceptualization, Writing – original draft, Writing – review & editing. AH: Data curation, Writing – original draft, Writing – review & editing. LR: Conceptualization, Data curation, Funding acquisition, Writing – original draft, Writing – review & editing.
