# Supplementary material for: Narrative analysis in individuals with Parkinson’s disease following intensive voice treatment: secondary outcome variables from a randomized controlled trial
Source: Front Hum Neurosci. 2024 May 22;18:1394948. doi: 10.3389/fnhum.2024.1394948 (PMC11150807; doi:10.3389/fnhum.2024.1394948)
Supplement: Supplementary file 1 [file Table_1.pdf]

# *Supplementary Material*

## 1.1 Supplementary Tables

| Block | ICC(2,1)>.90 (Excellent) | ICC(2,1)= .75-.90 (Good) | ICC(2,1)<.75 (Moderate) |
|---|---|---|---|
| **1** | TTR, total utterances, words, %nouns, %prepositions | Density, verbs per utterance, %adjective | %verb, %adverb |
| **2** | TTR, total utterances, words, %nouns | Density, %verbs, %prepositions, %adjectives | Verbs per utterance, %adverbs |
| **3** | TTR, density, verbs per utterance, total utterances, words, %verbs, %nouns, %prepositions, %adjectives | | %adverbs |
| **4** | TTR, total utterances, words, %verbs, %nouns, %prepositions | Verbs per utterance, %adjective, %adverb | Density |
| **Total** | TTR, total utterances, words, %nouns, %prepositions | Density, verbs per utterance, %verbs, % adjective, %adverbs | |

**Supplemental Table 1**. Interrater reliability, using intra-class coefficient (ICC), for EVAL.

frontiers
