# Supplementary material for: Narrative analysis in individuals with Parkinson’s disease following intensive voice treatment: secondary outcome variables from a randomized controlled trial
Source: Front Hum Neurosci. 2024 May 22;18:1394948. doi: 10.3389/fnhum.2024.1394948 (PMC11150807; doi:10.3389/fnhum.2024.1394948)
Supplement: Supplementary file 2 [file Table_2.pdf]

frontiers

Supplementary Material

Domain	Variable	TXPD	UNTXPD	Control
n		19	20	20
Loudness	dB SPL [a]			
	Mean (± SE)	69.7 ± 0.67	69.4 ± 0.66	71.3 ± 0.5
	Median	69.9	69.4	71.7
	Range	10.9	10.64	9.8
	Variance	8.42	8.7	5.6
Fluency and Efficiency	Words Per Minute			
	Mean (± SE)	126 ± 6	136 ± 7	136 ± 4
	Median	130	135	134
	Range	98	111	79
	Variance	631	971	368
	Number of Utterances			
	Mean (± SE)	13 ± .7	12 ± .8	14 ± 0.8
	Median	12	12	14
	Range	10	13	14
	Variance	9	13	13
Syntax	Verbs Per Utterance [c*]			

	Mean (± SE)	1.46 ± .07	1.81 ± .08	1.49 ± 0.08
	Median	1.46	1.74	1.36
	Range	1.15	1.28	1.53
	Variance	.01	.13	0.12
	Mean Length of Utterance			
	Mean (± SE)	10 ± .56	11 ± .49	10 ± 0.44
	Median	10	10	10
	Range	8	8	7
	Variance	10	5	4
Lexical-Semantic	Type-Token Ratio			
	Mean (± SE)	0.53 ± 0.01	0.54 ± 0.01	0.56 ± 0.01
	Median	0.54	0.54	0.53
	Range	0.18	0.23	0.15
	Variance	0.003	0.004	0.002
	Proposition Density [b,c*]			
	Mean (± SE)	.41 ± 0.01	0.45 ± 0.01	0.42 ± 0.01
	Median	0.42	0.46	0.42
	Range	0.18	0.14	0.10
	Variance	0.002	0.001	0.001

Informativeness	Main Concepts			
	Mean (± SE)	17 ± 0.50	17 ± 0.70	18 ± 0.62
	Median	17	17	17
	Range	8	11	9
	Variance	4	9	8
	Content Units			
	Mean (± SE)	19 ± 0.6	18 ± .9	20 ± 1
	Median	20	19	20
	Range	10	13	16
	Variance	7	17	19

Supplemental Table 2. Speech and language variables analyzed for the Cookie Theft picture in Controls and PD at baseline.

[a] Control > PD

[b] PD > Control

[c] UNTXPD > TXPD = Control

[d] Control > TXPD

[e] TXPD > Control

[f] TXPD = UNTXPD > Control

*$p < 0.01$
