# Supplementary material for: Narrative analysis in individuals with Parkinson’s disease following intensive voice treatment: secondary outcome variables from a randomized controlled trial
Source: Front Hum Neurosci. 2024 May 22;18:1394948. doi: 10.3389/fnhum.2024.1394948 (PMC11150807; doi:10.3389/fnhum.2024.1394948)
Supplement: Supplementary file 3 [file Table_3.pdf]

# *Supplementary Material*

| | | Fluency and Efficiency | | Syntax | | Lexical-Semantic | | Informativeness | |
|---|---|---|---|---|---|---|---|---|---|
| | SPL | WPM | # Utts | VPU | MLU | TTR | Density | MC | CU |
| Loudness | 1 | -0.254 | -0.244 | 0.134 | 0.13 | .332* | -0.177 | 0.029 | -0.156 |
| Words per Minute | -0.254 | 1 | .633** | -0.043 | 0.162 | -.587* | 0.233 | 0.204 | .599* |
| Number of Utterances | -0.244 | .633* | 1 | -.501* | -.491* | -.573* | 0.007 | 0.087 | .449* |
| Verbs per Utterance | 0.134 | -0.043 | -.501* | 1 | .619* | .312* | .376* | 0.201 | -0.218 |
| Mean Length of Utterance | 0.13 | 0.162 | -.491* | .619* | 1 | 0.135 | .400* | 0.12 | 0.074 |
| Type-Token Ratio | .332* | -.587* | -.573* | .312* | 0.135 | 1 | -0.065 | -0.061 | -.520* |
| Propositional Idea Density | -0.177 | 0.233 | 0.007 | .376* | .400* | -0.065 | 1 | 0.151 | 0.161 |
| Main Concepts | 0.029 | 0.204 | 0.087 | 0.201 | 0.12 | -0.061 | 0.151 | 1 | 0.244 |
| Content Units | -0.156 | .599* | .449* | -0.218 | 0.074 | -.520* | 0.161 | 0.244 | 1 |

**Supplemental Table 3**. Correlation matrix for the study variables at Baseline. SPL = sound pressure level for loudness, WPM = words per minute, # Utts = number of utterances, VPU = verbs per utterance, MLU = mean length of utterance, TTR = type-token ratio, MC = main concepts, CU = content units, Density = proposition density. * $p < .05$, ** $p < 0.01$.
