# Supplementary material for: Narrative analysis in individuals with Parkinson’s disease following intensive voice treatment: secondary outcome variables from a randomized controlled trial
Source: Front Hum Neurosci. 2024 May 22;18:1394948. doi: 10.3389/fnhum.2024.1394948 (PMC11150807; doi:10.3389/fnhum.2024.1394948)
Supplement: Supplementary file 4 [file Table_4.pdf]

Supplementary Material

	Quantile														
	**0.10**			**0.25**			**0.50**			**0.75**			**0.90**		
**Comparison**	**Estimate**	***t***	***p***	**Estimate**	***t***	***p***	**Estimate**	***t***	***p***	**Estimate**	***t***	***p***	**Estimate**	***t***	***p***
Number of Utterances															
Control Baseline - TXPD Baseline	0.86	0.70	0.864	2.36	2.53	0.181	2.43	2.38	0.151	1.81	1.70	0.486	0.86	0.70	0.864
Control Baseline - UNTXPD Baseline	2.51	2.01	0.600	2.52	2.06	0.207	2.05	1.97	0.238	1.33	1.24	0.529	2.51	2.01	0.600
Control Baseline - Control Follow-up	1.11	1.39	0.615	1.27	1.63	0.394	1.19	1.68	0.238	1.34	1.70	0.486	1.11	1.39	0.615
Control Baseline - TXPD Follow-up	0.42	0.34	0.923	2.51	2.29	0.181	2.79	2.36	0.151	2.32	1.67	0.486	0.42	0.34	0.923
Control Baseline - UNTXPD Follow-up	0.91	0.61	0.864	1.36	0.87	0.642	0.74	0.73	0.640	0.31	0.30	0.823	0.91	0.61	0.864
TXPD Baseline - UNTXPD Baseline	1.66	1.28	0.615	0.16	0.14	0.996	-0.38	-0.44	0.665	-0.47	-0.40	0.798	1.66	1.28	0.615
TXPD Baseline - Control Follow-up	0.26	0.21	0.944	-1.09	-1.11	0.593	-1.24	-1.16	0.411	-0.46	-0.41	0.798	0.26	0.21	0.944
TXPD Baseline - TXPD Follow-up	-0.44	-0.64	0.864	0.15	0.24	0.996	0.37	0.48	0.665	0.51	0.45	0.798	-0.44	-0.64	0.864
TXPD Baseline - UNTXPD Follow-up	0.06	0.04	0.967	-1.00	-0.68	0.680	-1.69	-1.61	0.238	-1.50	-1.16	0.529	0.06	0.04	0.967

UNTXPD Baseline - Control Follow-up	-1.40	-1.11	0.676	-1.24	-0.99	0.607	-0.86	-0.80	0.640	0.01	0.01	0.994	-1.40	-1.11	0.676
UNTXPD Baseline - TXPD Follow-up	-2.10	-1.57	0.600	-0.01	-0.01	0.996	0.75	0.65	0.647	0.99	0.64	0.783	-2.10	-1.57	0.600
UNTXPD Baseline - UNTXPD Follow-up	-1.60	-1.74	0.600	-1.16	-1.43	0.471	-1.31	-1.75	0.238	-1.02	-0.99	0.612	-1.60	-1.74	0.600
Control Follow-up - TXPD Follow-up	-0.70	-0.56	0.864	1.24	1.09	0.593	1.61	1.34	0.342	0.98	0.74	0.764	-0.70	-0.56	0.864
Control Follow-up - UNTXPD Follow-up	-0.20	-0.15	0.944	0.09	0.05	0.996	-0.45	-0.43	0.665	-1.03	-1.24	0.529	-0.20	-0.15	0.944
TXPD Follow-up - UNTXPD Follow-up	0.50	0.35	0.923	-1.15	-0.79	0.649	-2.05	-1.68	0.238	-2.01	-1.42	0.529	0.50	0.35	0.923
Mean length of utterance															
Control Baseline - TXPD Baseline	1.08	1.39	0.314	0.70	0.80	0.795	0.29	0.37	0.881	-0.31	-0.34	0.790	0.03	0.04	0.997
Control Baseline - UNTXPD Baseline	-0.86	-1.53	0.314	-0.60	-0.98	0.795	-0.76	-1.12	0.663	-0.66	-0.75	0.757	-0.81	-0.88	0.659
Control Baseline - Control Follow-up	-0.50	-0.94	0.477	-0.57	-1.09	0.795	-0.93	-1.40	0.663	-1.09	-1.61	0.453	-0.81	-1.15	0.659
Control Baseline - TXPD Follow-up	0.42	0.56	0.669	-0.26	-0.29	0.912	-1.00	-1.32	0.663	-1.76	-1.56	0.453	-1.41	-1.12	0.659
Control Baseline - UNTXPD Follow-up	-0.68	-1.28	0.339	-0.10	-0.21	0.912	-0.57	-0.92	0.671	0.16	0.19	0.848	0.67	0.67	0.750
TXPD Baseline - UNTXPD Baseline	-1.95	-2.63	0.148	-1.29	-1.38	0.795	-1.05	-1.13	0.663	-0.35	-0.41	0.790	-0.84	-0.85	0.659
TXPD Baseline - Control Follow-up	-1.58	-1.80	0.314	-1.27	-1.41	0.795	-1.22	-1.21	0.663	-0.78	-0.81	0.757	-0.83	-0.91	0.659
TXPD Baseline - TXPD Follow-up	-0.66	-1.41	0.314	-0.96	-1.75	0.795	-1.29	-2.16	0.496	-1.45	-2.02	0.453	-1.44	-1.47	0.591

TXPD Baseline - UNTXPD Follow-up	-1.76	-2.31	0.172	-0.80	-1.11	0.795	-0.86	-0.99	0.671	0.47	0.51	0.790	0.64	0.59	0.750
UNTXPD Baseline - Control Follow-up	0.36	0.63	0.663	0.03	0.04	0.969	-0.17	-0.23	0.881	-0.43	-0.44	0.790	0.00	0.00	0.997
UNTXPD Baseline - TXPD Follow-up	1.29	1.70	0.314	0.33	0.33	0.912	-0.24	-0.29	0.881	-1.10	-0.91	0.757	-0.60	-0.46	0.750
UNTXPD Baseline - UNTXPD Follow-up	0.19	0.45	0.699	0.50	0.83	0.795	0.19	0.33	0.881	0.82	1.14	0.645	1.48	1.66	0.591
Control Follow-up - TXPD Follow-up	0.92	1.19	0.352	0.31	0.32	0.912	-0.07	-0.08	0.936	-0.67	-0.65	0.779	-0.61	-0.47	0.750
Control Follow-up - UNTXPD Follow-up	-0.18	-0.31	0.757	0.47	0.68	0.826	0.36	0.58	0.856	1.25	1.44	0.463	1.48	1.42	0.591
TXPD Follow-up - UNTXPD Follow-up	-1.10	-1.47	0.314	0.16	0.19	0.912	0.43	0.57	0.856	1.92	1.67	0.453	2.08	1.60	0.591
Type-Token Ratio															
Control Baseline - TXPD Baseline	0.02	1.23	0.893	0.06	2.18	0.137	0.00	-0.09	0.926	0.00	0.03	0.979	-0.03	-0.78	0.821
Control Baseline - UNTXPD Baseline	0.00	-0.02	0.994	0.03	1.26	0.348	-0.02	-0.55	0.926	-0.03	-1.00	0.931	-0.04	-1.01	0.676
Control Baseline - Control Follow-up	0.00	-0.08	0.994	0.00	-0.19	0.849	0.01	0.68	0.926	0.01	0.60	0.931	0.02	1.80	0.676
Control Baseline - TXPD Follow-up	0.02	0.93	0.893	0.07	2.45	0.118	-0.01	-0.19	0.926	0.00	-0.03	0.979	-0.04	-0.65	0.861
Control Baseline - UNTXPD Follow-up	0.00	0.04	0.994	0.02	0.95	0.430	-0.01	-0.31	0.926	-0.02	-0.55	0.931	-0.01	-0.27	0.924
TXPD Baseline - UNTXPD Baseline	-0.02	-1.32	0.893	-0.03	-1.00	0.430	-0.01	-0.41	0.926	-0.03	-0.89	0.931	-0.01	-0.13	0.963
TXPD Baseline - Control Follow-up	-0.02	-1.23	0.893	-0.06	-2.12	0.137	0.01	0.45	0.926	0.01	0.37	0.931	0.05	1.30	0.676
TXPD Baseline - TXPD Follow-up	0.00	0.01	0.994	0.01	0.59	0.598	0.00	-0.17	0.926	0.00	-0.07	0.979	-0.01	-0.25	0.924

TXPD Baseline - UNTXPD Follow-up	-0.02	-0.81	0.893	-0.04	-1.32	0.348	-0.01	-0.24	0.926	-0.02	-0.53	0.931	0.02	0.48	0.861
UNTXPD Baseline - Control Follow-up	0.00	-0.04	0.994	-0.03	-1.40	0.348	0.02	0.79	0.926	0.04	1.10	0.931	0.06	1.61	0.676
UNTXPD Baseline - TXPD Follow-up	0.02	0.91	0.893	0.04	1.45	0.348	0.01	0.26	0.926	0.03	0.70	0.931	0.00	-0.01	0.991
UNTXPD Baseline - UNTXPD Follow-up	0.00	0.08	0.994	-0.01	-0.74	0.534	0.00	0.26	0.926	0.01	0.60	0.931	0.03	1.33	0.676
Control Follow-up - TXPD Follow-up	0.02	1.14	0.893	0.07	2.45	0.118	-0.01	-0.48	0.926	-0.01	-0.33	0.931	-0.06	-1.04	0.676
Control Follow-up - UNTXPD Follow-up	0.00	0.08	0.994	0.02	1.11	0.406	-0.02	-0.51	0.926	-0.03	-0.72	0.931	-0.03	-1.11	0.676
TXPD Follow-up - UNTXPD Follow-up	-0.02	-0.71	0.893	-0.05	-1.77	0.237	-0.01	-0.14	0.926	-0.02	-0.44	0.931	0.03	0.50	0.861
Verbs per Utterance															
Control Baseline - TXPD Baseline	0.06	0.76	0.561	0.07	0.60	0.593	-0.01	-0.09	0.947	0.01	-0.09	0.966	-0.14	-0.93	0.533
Control Baseline - UNTXPD Baseline	-0.27	-2.63	**0.040**	-0.33	-2.73	**0.037**	-0.38	-2.52	0.105	-0.39	-3.12	**0.017**	-0.36	-2.87	**0.037**
Control Baseline - Control Follow-up	0.00	0.02	0.996	0.00	-0.05	0.957	0.01	0.07	0.947	-0.02	-0.16	0.966	-0.05	-0.34	0.785
Control Baseline - TXPD Follow-up	0.00	0.03	0.996	-0.13	-1.00	0.481	-0.17	-1.37	0.412	-0.24	-1.02	0.579	-0.50	-1.88	0.189
Control Baseline - UNTXPD Follow-up	-0.15	-1.51	0.252	-0.21	-2.15	0.101	-0.12	-1.13	0.412	-0.05	-0.42	0.966	0.02	0.16	0.871
TXPD Baseline - UNTXPD Baseline	-0.33	-4.00	**0.002**	-0.39	-3.75	**0.004**	-0.37	-2.28	0.122	-0.38	-2.41	0.065	-0.22	-1.36	0.331
TXPD Baseline - Control Follow-up	-0.06	-0.80	0.561	-0.07	-0.84	0.501	0.02	0.14	0.947	-0.01	-0.04	0.966	0.09	0.46	0.753

TXPD Baseline - TXPD Follow-up	-0.06	-0.83	0.561	-0.20	-1.95	0.114	-0.16	-1.10	0.412	-0.22	-1.13	0.579	-0.36	-1.48	0.307
TXPD Baseline - UNTXPD Follow-up	-0.21	-2.51	**0.041**	-0.28	-2.28	0.093	-0.11	-0.96	0.460	-0.03	-0.29	0.966	0.16	1.02	0.518
UNTXPD Baseline - Control Follow-up	0.27	2.60	**0.040**	0.32	3.21	**0.013**	0.39	2.50	0.105	0.37	2.64	**0.048**	0.31	1.61	0.276
UNTXPD Baseline - TXPD Follow-up	0.27	2.61	**0.040**	0.19	1.34	0.341	0.21	1.22	0.412	0.15	0.78	0.731	-0.14	-0.55	0.753
UNTXPD Baseline - UNTXPD Follow-up	0.12	1.02	0.519	0.11	0.85	0.501	0.25	2.06	0.157	0.34	4.04	**0.002**	0.38	3.26	**0.023**
Control Follow-up - TXPD Follow-up	0.00	0.01	0.996	-0.13	-1.09	0.461	-0.18	-1.41	0.412	-0.22	-1.08	0.579	-0.45	-1.97	0.189
Control Follow-up - UNTXPD Follow-up	-0.15	-1.66	0.250	-0.21	-2.04	0.109	-0.13	-1.25	0.412	-0.03	-0.26	0.966	0.07	0.45	0.753
TXPD Follow-up - UNTXPD Follow-up	-0.15	-1.58	0.250	-0.08	-0.60	0.593	0.04	0.41	0.855	0.19	1.03	0.579	0.52	2.15	0.169
Propositional Idea Density															
Control Baseline - TXPD Baseline	-0.01	-0.14	0.906	0.01	0.07	0.932	0.00	0.08	0.980	-0.04	0.08	0.811	-0.03	0.07	0.688
Control Baseline - UNTXPD Baseline	0.16	2.22	0.132	0.15	0.07	0.318	0.15	0.07	0.257	0.11	0.08	0.518	0.16	0.07	0.212
Control Baseline - Control Follow-up	0.07	1.75	0.291	0.08	0.04	0.318	-0.01	0.07	0.980	0.01	0.05	0.832	0.06	0.04	0.342
Control Baseline - TXPD Follow-up	0.11	1.53	0.291	0.06	0.08	0.634	0.09	0.07	0.405	0.03	0.08	0.832	0.11	0.09	0.342
Control Baseline - UNTXPD Follow-up	0.10	1.42	0.291	0.09	0.07	0.528	0.09	0.07	0.405	0.05	0.08	0.811	0.12	0.07	0.320
TXPD Baseline - UNTXPD Baseline	0.17	2.29	0.132	0.15	0.07	0.318	0.15	0.08	0.257	0.15	0.07	0.518	0.20	0.08	0.180
TXPD Baseline - Control Follow-up	0.08	1.10	0.409	0.07	0.08	0.528	-0.02	0.10	0.980	0.06	0.08	0.811	0.09	0.07	0.342

TXPD Baseline - TXPD Follow-up	0.12	2.74	0.093	0.05	0.05	0.528	0.09	0.05	0.257	0.08	0.04	0.518	0.15	0.08	0.212
TXPD Baseline - UNTXPD Follow-up	0.11	1.52	0.291	0.08	0.07	0.528	0.09	0.08	0.405	0.09	0.07	0.522	0.15	0.08	0.212
UNTXPD Baseline - Control Follow-up	-0.09	-1.24	0.356	-0.07	0.07	0.528	-0.17	0.09	0.257	-0.10	0.07	0.518	-0.10	0.07	0.342
UNTXPD Baseline - TXPD Follow-up	-0.05	-0.67	0.687	-0.10	0.08	0.528	-0.07	0.07	0.495	-0.08	0.07	0.624	-0.05	0.09	0.687
UNTXPD Baseline - UNTXPD Follow-up	-0.06	-1.47	0.291	-0.07	0.04	0.318	-0.06	0.04	0.315	-0.06	0.04	0.518	-0.04	0.04	0.470
Control Follow-up - TXPD Follow-up	0.04	0.56	0.720	-0.02	0.08	0.900	0.10	0.09	0.405	0.02	0.07	0.832	0.05	0.09	0.685
Control Follow-up - UNTXPD Follow-up	0.03	0.44	0.758	0.01	0.07	0.932	0.10	0.09	0.405	0.04	0.07	0.811	0.06	0.07	0.577
TXPD Follow-up - UNTXPD Follow-up	-0.01	-0.12	0.906	0.03	0.08	0.887	0.00	0.07	0.980	0.02	0.07	0.832	0.00	0.09	0.957

Supplemental Table 4. Comparisons across groups and visits by quantiles. Bold: $p_{FDR} < 0.05$.
