# Supplementary material for: Narrative analysis in individuals with Parkinson’s disease following intensive voice treatment: secondary outcome variables from a randomized controlled trial
Source: Front Hum Neurosci. 2024 May 22;18:1394948. doi: 10.3389/fnhum.2024.1394948 (PMC11150807; doi:10.3389/fnhum.2024.1394948)
Supplement: Supplementary file 5 [file Data_Sheet_1.pdf]

Supplementary Material

1 Supplementary Material

1.1 LSVT Intervention

The first 30 minutes of each treatment session targeted: (*a*) a minimum of 15 maximum duration sustained vowels (e.g., "say 'ah' for as long as you can using your loud voice"); (*b*) a minimum of 15 maximum f_0 range (e.g., "say 'ah' as high/low as you can with your loud or big voice"); and (*c*) a minimum of 5 repetitions of 10 functional phrases selected by participants as phrases used in their daily communication. The second 30 minutes of each treatment session included individualized speech hierarchies designed to maximize functional communication goals. Speech hierarchies progressed both in length of utterance (words, phrases, sentences, etc.) and task complexity (repetition, reading, conversation, communication activities, with/without distractors). Further, speech hierarchy practice materials were made meaningful to each participant by selecting topics of interest, hobbies or activities related to functional communication goals for each participant. For example, in week 1 of treatment, the tasks might include structured reading at the word/phrase level and spontaneous single word/phrase level responses to questions, word association tasks, fill-in-the-blank or simple descriptions. In week 2, the target would increase to structured reading at the sentence level and spontaneous short/simple conversation. Week 3 might involve structured reading or spontaneous conversation at the paragraph level; week 4 might target continuous conversation. During all these tasks, participants were required to maintain their target loudness giving maximum effort. Further, for each participant, speech hierarchy practice materials were made meaningful by selecting topics of interest, hobbies, or activities related to functional communication goals. Throughout the entire one-hour session, focus on sensory awareness and functional goals were emphasized to encourage generalization (e.g., "Feel that effort when you use your loud voice? That is

what you need to feel when you answer the phone at work.”). All participants were required to do homework once per day on the day of treatment and twice per day on days when treatment did not occur. Homework involved repetitions of the same three exercises from the first 30 minutes of treatment sessions, as well as hierarchy practice and carry over assignments (i.e., ordering dinner at a restaurant using the target louder voice) (see Fox et al., 2012 and Ramig et al., 2018 for the full protocol).

To ensure treatment fidelity, three LSVT LOUD certified speech-language pathologists administered treatment following established protocols, including instructions about encouragement and positive reinforcement during treatment, ensuring treatment fidelity. Additional information about the training of clinicians, control of bias, and maintaining treatment fidelity are presented in Levy et al. (2020) and Ramig et al. (2018).

1. Fox C, Ebersbach G, Ramig L, Sapir S. LSVT LOUD and LSVT BIG: Behavioral Treatment Programs for Speech and Body Movement in Parkinson Disease. Parkinsons Dis. 2012;2012:391946.
2. Levy ES, Moya-Galé G, Chang YHM, Freeman K, Forrest K, Brin MF, et al. The effects of intensive speech treatment on intelligibility in Parkinson’s disease: A randomised controlled trial. EClinicalMedicine. 2020 Jul 1;24:100429.
3. Ramig L, Halpern A, Spielman J, Fox C, Freeman K. Speech treatment in Parkinson’s disease: Randomized controlled trial (RCT). Movement Disorders. 2018;33(11):1777–91.
