# Supplementary material for: Narrative analysis in individuals with Parkinson’s disease following intensive voice treatment: secondary outcome variables from a randomized controlled trial
Source: Front Hum Neurosci. 2024 May 22;18:1394948. doi: 10.3389/fnhum.2024.1394948 (PMC11150807; doi:10.3389/fnhum.2024.1394948)
Supplement: Supplementary file 6 [file Data_Sheet_2.pdf]

Supplementary Material

1 Supplementary Material

1.2 Main Concept Analysis and Content Units

Accurate and complete productions had to include each element of the main concept, allowing for alternative productions if they conveyed the same meaning. For example, the statement, "the little **boy** that's up **on the stool** that's about ready **to fall**," was considered accurate and complete for two main concepts: #3 "the [1]**boy** is [2]**on** a [3]**stool**" and #5 "the [1]**stool** is [2]**tipping**" (where numbered/bolded words indicate essential elements). As illustrated in this example as well as in additional examples from Appendix B of Nicholas and Brookshire (1995, p. 155-156), utterances that contained two main concepts were broken up according to the following rules: 1) each main concept should have a subject, verb, and all necessary objects and 2) if the utterance contains only one subject but two verbs separated by "and," the subject may be reused. At times, prepositional phrases were divided from utterances with a single subject and verb to allow participants to receive partial credit for more than one main concept. If a participant produced more than one utterance that could be matched with a single main concept, the utterance that was the most accurate and complete was coded (as opposed to coding the final version as originally suggested by Nicholas & Brookshire, 1995). For each main concept that was present, the participant's matching utterance was coded as accurate and complete (AC, 3 points), accurate but incomplete (AI, 2 points), inaccurate but complete (IC, 2 points), or inaccurate and incomplete (II, 1 point). Absent main concepts (AB) received a score of 0 points.

In addition to main concept analysis, Yorkston and Beukelman's (1980) 56 content units for the Cookie Theft picture were counted for each participant, accounting for alternative wordings. In this coding system, if participants mentioned a content unit twice, only one use was counted. Additionally, if a participant's utterance matched two content units, only one was counted.

Each of the transcribers/coders completed three training examples to learn main concept analysis and coding of content units. Disagreements were discussed and resolved with the lead researchers (AER and KJG). For main concept analysis, reliability training continued until interrater reliability was deemed adequate (kappa > 0.75, point-to-point > 0.8) for at least 80% of transcripts in a set (12 of 15 transcripts met this criterion in the final set; note that these 15 samples were not counted toward the reliability sample, as they contributed to training). For content units, reliability was excellent (kappa and point-to-point > 0.9) for all training examples.

1. Yorkston KM, Beukelman DR. An analysis of connected speech samples of aphasic and normal speakers. The Journal of Speech and Hearing Disorders. 1980 Feb;45(1):27–36.
